# P096: Antibiotic stewardship in intensive care units: a cross sectional study of 355 ICUs in Germany

**DOI:** 10.1186/2047-2994-2-S1-P96

**Published:** 2013-06-20

**Authors:** F Maechler, F Schwab, E Meyer, C Geffers, R Leistner, P Gastmeier

**Affiliations:** 1 Infection Control, Charité Berlin, Berlin, Germany

## Introduction

Little information is available on antibiotic prescription management in German hospitals.

## Objectives

The objective of this cross sectional study was to determine the prevalence and components of antibiotic stewardship (ABS) measures in German ICUs.

## Methods

A questionnaire survey was sent to all ICU-members of the German nosocomial infection surveillance system KISS (n=579) in October 2011. Data on antibiotic management structures were collected and analyzed by structural hospital and ICU factors.

## Results

The questionnaire was completed by 355 German ICUs (61% response rate).

The most common measures used (>80% of the ICUs) were personal restrictions for antibiotic prescriptions, routine access to unit-based bacterial resistance data and pharmacy reports on antibiotic costs and consumption. A small proportion of ICUs (14%) employed ABS consultants or infectious diseases specialists for the prescription of antimicrobial medication. Hospitals with their own integrated microbiological laboratory report twice as much to take part in surveillance networks of antimicrobial use (34%) and bacterial resistance (32%) compared with hospitals with external laboratories (15% and 14%, respectively, p<0.001). Also, non-profit and public hospitals participate more often in surveillance systems for bacterial resistance than private hospitals (>23% vs. 11%, p<0.05).

## Conclusion

While the majority of ICUs report to have some antibiotic policies established, the contents and composition of these strategies vary markedly. Organizational-leveled control strategies to improve antibiotic control are already quite common in Germany. In contrast, other strategies widely considered effective such as the continuous surveillance of antimicrobial use and bacterial resistance or the employment of infectious disease consultants are still scarce. More effort is required with regard to incentives for infectious diseases consultants and to the participation in surveillance networks.

This study provides a benchmark for future antibiotic stewardship programs.

## Disclosure of interest

None declared

